# Applicability of ChatGPT in Assisting to Solve Higher Order Problems in Pathology

**DOI:** 10.7759/cureus.35237

**Published:** 2023-02-20

**Authors:** Ranwir K Sinha, Asitava Deb Roy, Nikhil Kumar, Himel Mondal

**Affiliations:** 1 Pathology, All India Institute of Medical Sciences, Deoghar, Jharkhand, IND; 2 Physiology, All India Institute of Medical Sciences, Deoghar, Jharkhand, IND

**Keywords:** critical reasoning, intelligence, cognition, decision making, students, microcomputers, problem-solving, artificial intelligence, chatgpt, pathologists

## Abstract

Background

Artificial intelligence (AI) is evolving for healthcare services. Higher cognitive thinking in AI refers to the ability of the system to perform advanced cognitive processes, such as problem-solving, decision-making, reasoning, and perception. This type of thinking goes beyond simple data processing and involves the ability to understand and manipulate abstract concepts, interpret, and use information in a contextually relevant way, and generate new insights based on past experiences and accumulated knowledge. Natural language processing models like ChatGPT is a conversational program that can interact with humans to provide answers to queries.

Objective

We aimed to ascertain the capability of ChatGPT in solving higher-order reasoning in the subject of pathology.

Methods

This cross-sectional study was conducted on the internet using an AI-based chat program that provides free service for research purposes. The current version of ChatGPT (January 30 version) was used to converse with a total of 100 higher-order reasoning queries. These questions were randomly selected from the question bank of the institution and categorized according to different systems. The responses to each question were collected and stored for further analysis. The responses were evaluated by three expert pathologists on a zero to five scale and categorized into the structure of the observed learning outcome (SOLO) taxonomy categories. The score was compared by a one-sample median test with hypothetical values to find its accuracy.

Result

A total of 100 higher-order reasoning questions were solved by the program in an average of 45.31±7.14 seconds for an answer. The overall median score was 4.08 (Q1-Q3: 4-4.33) which was below the hypothetical maximum value of five (one-test median test p <0.0001) and similar to four (one-test median test p = 0.14). The majority (86%) of the responses were in the “relational” category in the SOLO taxonomy. There was no difference in the scores of the responses for questions asked from various organ systems in the subject of Pathology (Kruskal Wallis p = 0.55). The scores rated by three pathologists had an excellent level of inter-rater reliability (ICC = 0.975 [95% CI: 0.965-0.983]; F = 40.26; p < 0.0001).

Conclusion

The capability of ChatGPT to solve higher-order reasoning questions in pathology had a relational level of accuracy. Hence, the text output had connections among its parts to provide a meaningful response. The answers from the program can score approximately 80%. Hence, academicians or students can get help from the program for solving reasoning-type questions also. As the program is evolving, further studies are needed to find its accuracy level in any further versions.

## Introduction

Artificial intelligence (AI) is evolving in healthcare and biomedical literature. AI has the potential to significantly impact the diagnosis of diseases by improving the accuracy, speed, and efficiency of decision-making. AI algorithms can process vast amounts of data, identify patterns, and make predictions that may be beyond the capabilities of human physicians [1]. One example of AI in diagnostic pathology is the use of deep learning algorithms to analyze medical images, such as histopathology slides, to identify and diagnose diseases. These algorithms can identify complex patterns and features in the images, such as the presence of cancerous cells, with high accuracy, reducing the likelihood of misdiagnosis [2]. Another application of AI in diagnostic pathology is the use of natural language processing (NLP) algorithms to analyze pathology reports, extract relevant information, and assist in disease diagnosis. NLP algorithms can identify key symptoms, comorbidities, and demographic information from pathology reports, helping pathologists to make more informed diagnoses [3].

Higher cognitive thinking in AI refers to the ability of AI systems to perform advanced cognitive processes, such as problem-solving, decision-making, reasoning, and perception. This type of thinking goes beyond simple data processing and involves the ability to understand and manipulate abstract concepts, interpret and use information in a contextually relevant way, and generate new insights based on past experiences and accumulated knowledge [4]. However, it still has some limitations as it lacks the human ability to think creatively, understand emotions, and exhibit ethical judgment [5].

The capability of AI in solving higher-order reasoning type of questions in the subject of pathology is dependent on the level of complexity of the questions and the training data that the AI system has been exposed to. For basic or straightforward questions, AI systems can provide accurate and relevant answers in real-time [6]. For example, a chatbot trained in pathology fundamentals could provide answers to questions related to anatomy and physiology, common diseases, and their symptoms. However, when it comes to more complex questions that require a deep understanding of pathology and medical knowledge, AI systems might not be as effective as human experts. For example, questions that require critical thinking, reasoning, and interpretation may be beyond the current capabilities of AI systems [7].

ChatGPT is one such AI-based conversational program that can generate human-like responses and it is on trial for biomedical writing [8]. The current version of the ChatGPTis free for research purposes. In this study, we aimed to ascertain the capability of ChatGPT in solving higher-order pathological reasoning.

## Materials and methods

Type, setting, and ethics

This was a cross-sectional study conducted in the first and second week of February 2023. The data for this study was collected from a free program available on the internet. We used personal computers and broadband internet connection for collecting the data. This study does not involve any human research participants. Hence, according to prevailing guidelines, the study does not require any institutional ethics review.

Tool

We used the current version (January 30, 2023) of ChatGPT (https://chat.openai.com) for generating the solution to higher-order reasoning in pathology. This version is for trial for the public and research purposes. ChatGPT is capable of responding to complex commands by using its advanced natural language processing capabilities and its vast training data to analyze and understand the input text. The model is able to generate relevant and meaningful responses to a wide range of questions and commands, including those that are complex in nature [9].

Questions

We randomly selected a total of 100 questions from the question bank of the department. Furthermore, we categorized the questions into 11 systems of pathology (e.g., general pathology, cardiovascular pathology, gastrointestinal pathology). The questions were of a higher order; the answer to the question requires an in-depth knowledge of the subject matter. It focuses on underlying concepts and principles, rather than just rote memorization of facts. For example, instead of asking to recall a definition, asking to apply a concept to a new situation require analysis and synthesis of knowledge [10]. The face and content validity of the questions were checked by an expert pathologist with teaching and research experience of > 10 years. The answer keys of the questions were pre-defined to make the assessment more objective in nature.

Data collection

The questions were used as input for the conversation with ChatGPT. The answer provided by the program was copied into a notepad. It was saved on the computer for further analysis. The data collection ranged from February 5 to February 10, 2023. The questions and collected text were then printed for evaluation by pathologists.

Scoring method

**Figure 1:**
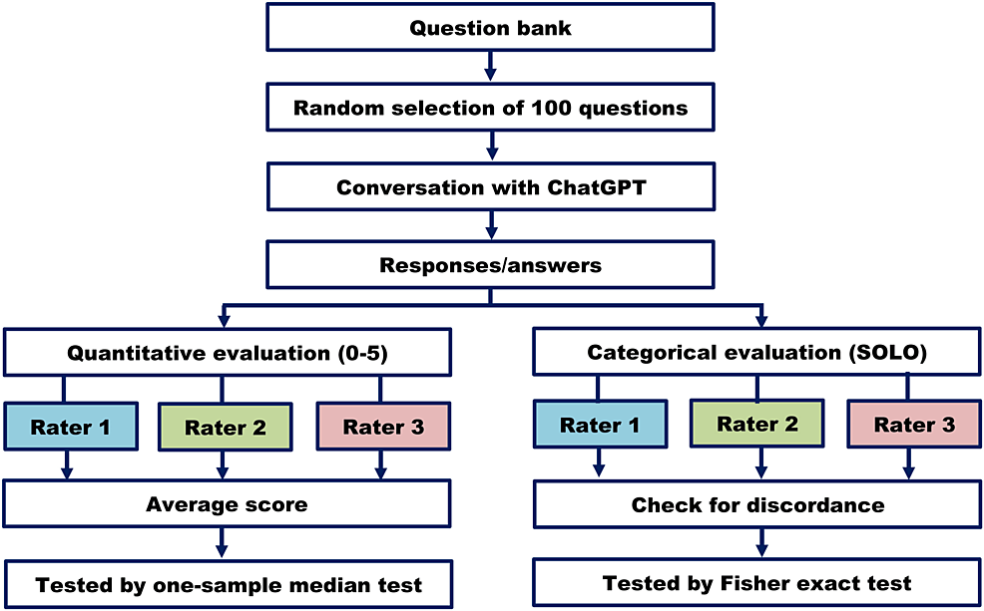
Brief study method flow chart SOLO: Structure of the Observed Learning Outcome

Two scoring methods were used. First, the answers were evaluated on a 0-5 scale by three pathologists according to the pre-selected answer key. Their ratings were stored individually for calculating the average score. Next, we used the structure of the observed learning outcome (SOLO) taxonomy for evaluating the answers. It is a framework for evaluating the quality and depth of individual learning. The answers are allotted to five categories - pre-structural (no understanding of the task), unistructural (limited understanding of the task), multistructural (understanding multiple aspects but no connection among them), relational (understanding connections and relationships between multiple aspects) and extended abstract (deep and sophisticated understanding incorporating abstract and theoretical concepts) [11]. A brief study method is shown in Figure 1.

Statistical analysis

We used descriptive statistical tests to express the data in number, mean, median, standard deviation, and first and third quartile. The data were not normally distributed (tested by the Shapiro-Wilk test). For checking the accuracy of the response, we used a one-sample median test with hypothetical expected values (e.g., a comparison with a hypothetical value of 4, if found not to be statistically significantly different, then the median score may be approximately four). The scores according to different systems of pathology were tested by Friedman’s test with a posthoc test. The score among the three raters was tested by the intraclass correlation coefficient (ICC). The categorical data were compared by Fisher’s exact test (as the frequency was found to be less than five in a category) [12]. We used GraphPad Prism 7 (GraphPad Software Inc., USA) for all the statistical analysis. We considered p-value < 0.05 as statistical significance.

## Results

Among the 100 responses, the overall median score was 4.08 (Q1-Q3: 4-4.33). The overall and system-wise scores of the responses are shown in Table 1.

**Table 1:** Overall and system-wise scores of the responses *The p-value of a one-sample median test with a hypothetical value of 5. A significant p-value indicates that the median score is far away from 5. A non-significant p-value indicates that the score is not different from 5 (if it is higher or lower, check the median column). †The p-value of a one-sample median test with a hypothetical value of 4. A significant p-value indicates that the median score is far away from 4. A non-significant p-value indicates that the score is not different from 4 (if it is higher or lower, check the median column). SD = standard deviation, Q1 = first quartile, Q3 = third quartile

Category	Mean±SD	Median (Q1-Q3)	95% confidence interval	P-value (hypothetical value 5)*	P-value (hypothetical value 4)†
Overall (n = 100)	4.01±0.61	4.08 (4-4.33)	3.89-4.13	<0.0001	0.14
General (n = 15)	3.92±0.47	4 (3.42-4.33)	3.66-4.18	<0.0001	0.37
Hematology (n = 16)	3.89±0.54	4 (3.79-4.33)	3.61-4.19	<0.0001	0.53
Respiratory (n = 9)	3.87±1.21	4.17 (4.17-4.33)	2.94-4.8	0.004	0.15
Gastrointestinal (n = 7)	4.19±0.22	4.17 (4-4.42)	3.98-4.4	0.02	0.13
Cardiovascular (n = 8)	4.25±0.2	4.25 (4.13-4.38)	4.08-4.42	0.008	0.03
Hepatobiliary (n = 5)	4.3±0.22	4.33 (4.17-4.5)	4.03-4.57	0.06	0.13
Genitourinary tract (n = 10)	3.8±1.12	4 (4-4.17)	3-4.6	0.002	0.69
Female genital tract and breast (n = 12)	4.13±0.38	4 (4-4.5)	3.89-4.36	0.0005	0.45
Endocrine (n = 7)	4.21±0.27	4 (4-4.5)	3.97-4.46	0.02	0.25
Musculoskeletal (n = 6)	4.17±0.41	4.25 (4-4.5)	3.74-4.6	0.03	0.63
Nervous System (n = 5)	3.97±2.27	4 (4-4.17)	3.63 -4.31	0.06	>0.99

This was significantly lower than the highest achievable score of 5 but similar to a score of 5. In hepatobiliary and nervous system pathology, the score was similar to 5. For the rest of the system, the score was similar to 4.

**Figure 2:**
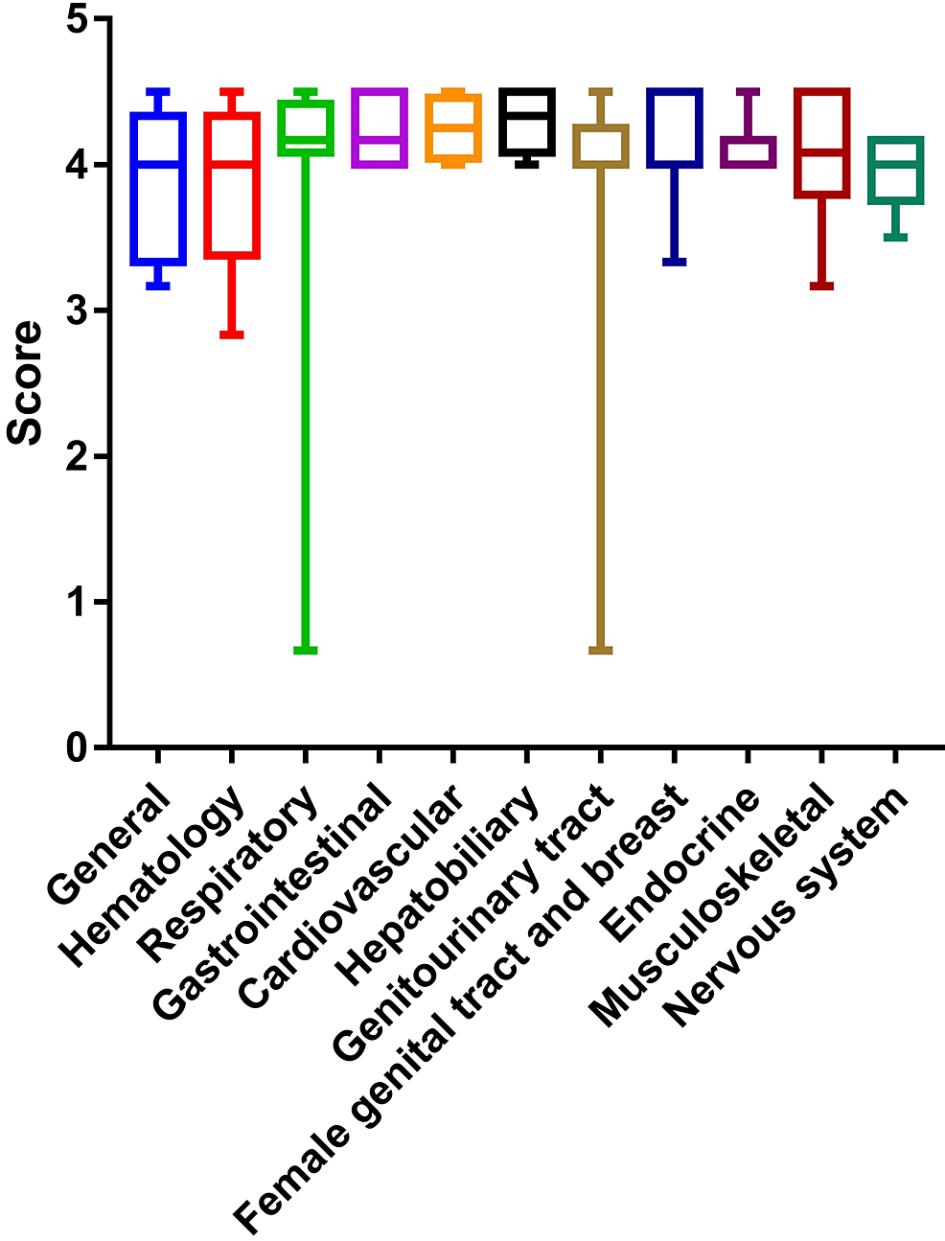
System-wise average scores of the responses

The scores according to various systems of pathology showed no significant difference in the Kruskal-Wallis test (p = 0.55), which is shown in Figure 2. As there was no significant difference, the posthoc test p-values were not presented.

**Figure 3:**
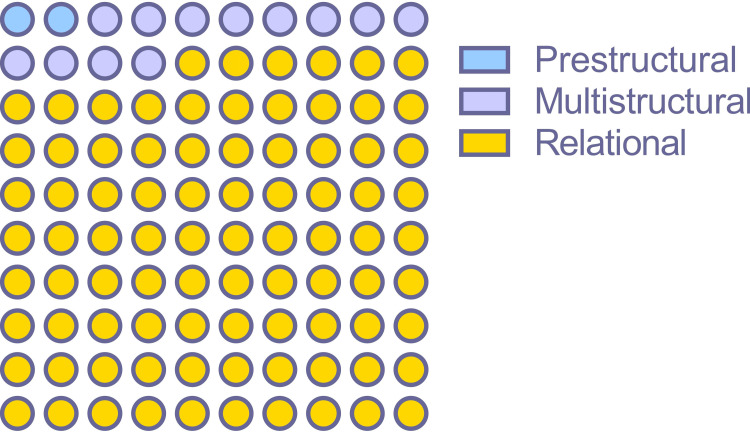
The category of response according to the structure of observed learning outcome taxonomy

A total of 86 responses were in the “relational” level, 12 were in “multistructural”, and two were in “prestructural” (p < 0.0001). The evaluation category-wise distribution of 100 responses is shown in Figure 3.

**Figure 4:**
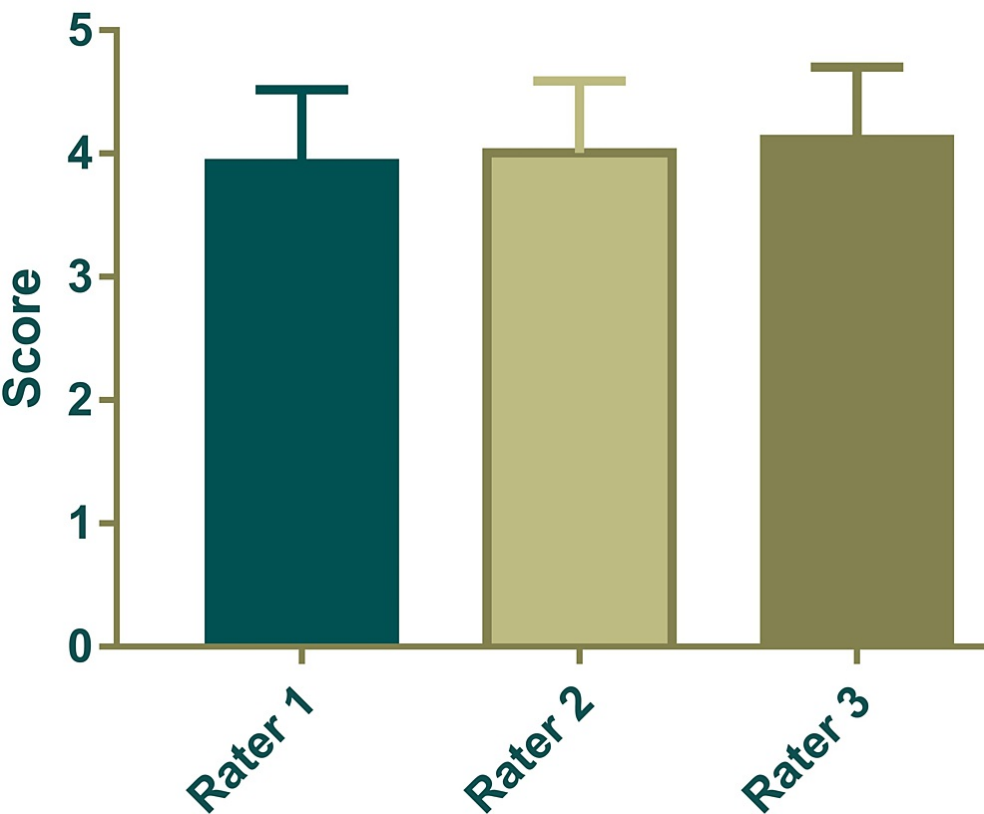
The scores of the responses (on a scale ranging from 0 to 5) by three raters

The score provided by the three rates has an excellent level of inter-rater reliability. The ICC was 0.975 with a 95% confidence interval of 0.965 to 0.983 (F = 40.26, p < 0.0001). The scores by raters are shown in Figure 4.

**Figure 5:**
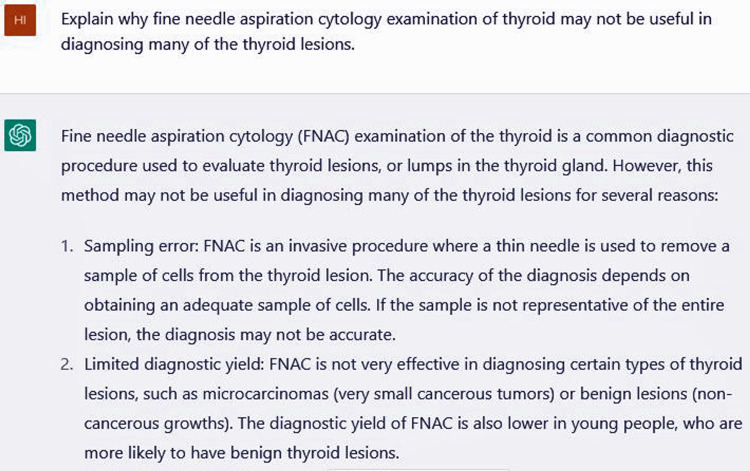
Screenshot showing part of a conversation with ChatGPT

**Figure 6:**
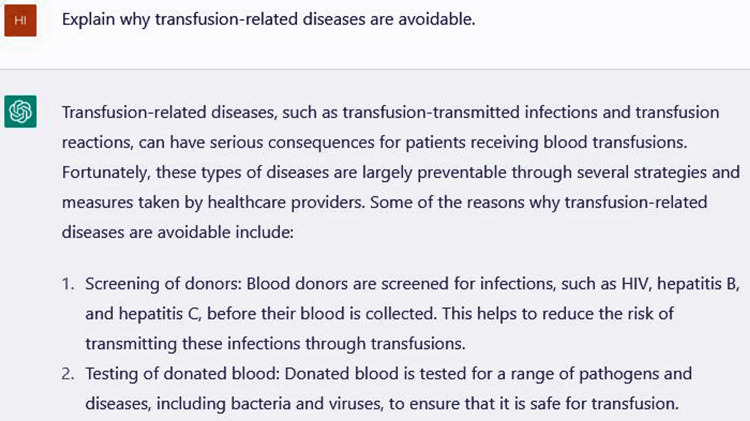
Screenshot showing a part of a conversation with ChatGPT

Two examples of conversation with ChatGPT (partial screenshot) are shown in Figures 5, 6.

## Discussion

To ascertain the capability of ChatGPT in solving higher-order reasoning in pathology, we used 100 random questions and found that the responses provided by ChatGPT is having a relational level of response with a score similar to four out of five.

Several previous studies have been conducted to check the capability of ChatGPT for medical educational applications. A study by Gilson et al. found that ChatGPT has the capability of answering medical questions using natural language processing which is similar to a third-year medical student in the United States. They also reported its capability to give reasoning and informative context throughout the majority of replies owing to the dialogic character of the response to inquiries [13]. In this study, we used the reasoning-type questions asked commonly to a second-third-year medical student studying in Indian medical colleges. The study result by Gilson and our study is corroborative. Another study by Kung et al. found that the ChatGPT has the capability to pass the United States Medical Licensing Examination without the help of any human. Furthermore, ChatGPT displayed comprehensible reasoning and valid clinical insights in the responses [14]. In contrast, a study by Huh found that ChatGPT’s ability to compete with a Korean student in parasitology is still low [15].

Hence, we suggest that medical schools and colleges may not restrict the use of AI but train the students to take advantage of it with judicial use. Furthermore, future AI systems must be carefully designed, developed, and validated to ensure they provide accurate and trustworthy information to medical students. Further development of AI especially for health-related information would enhance the capability of AI to be used in education and healthcare systems [16]. The current ChatGPT has limitations in that they have information on 2021. Hence, recent advances may not be available in its output. It is essential that AI systems are monitored and updated regularly to ensure they remain relevant and up to date with the latest advances in pathology and medical knowledge.

AI may be able to recognize patterns and classify data, but it lacks the ability to truly understand the underlying meaning and context of information. Although AI can process and analyze large amounts of data, it may not be able to identify the relationships between different pieces of information in any complex medical situation. AI cannot make subjective judgments or ethical evaluations, as it lacks the ability to understand personal values and biases [17]. AI may be able to generate new information based on existing data, but it cannot create truly original and innovative ideas without human input. Hence, in healthcare and medical education, careful use of technology is needed so that it can facilitate human decisions, not replace them [18].

Limitations

This study has several limitations. We used a scoring method ranging from 0 to 5. Although the answer keys were prepared beforehand, a subjective evaluation bias still may present which was beyond our control. Furthermore, the SOLO taxonomy categorization was also a subjective method of evaluation. We used questions from our question bank. Other institutions may have different collections of questions. Hence, in the future, a multicentric study may be conducted for a more generalizable result. A slight modification in question can generate a different response in ChatGPT. Hence, the response may be different if the question is paraphrased. These should be kept in consideration in future studies.

## Conclusions

The capability of ChatGPT to solve higher-order reasoning questions in pathology had a relational level of accuracy. Hence, the text output had connections among its parts to provide a meaningful response. This level of cognition in AI can help students and academicians to get a handy response to their queries. However, as AI, all over the world, AI programs are evolving. Hence, the capability of AI should be tested further in future studies.

## Appendices

Contribution details

Ranwir Kumar Sinha: Concept, Data collection, Literature search, Editing manuscript, Approving final version of the manuscript. Asitava Deb Roy: Concept, Data collection, Literature search, Editing manuscript, Approving final version of the manuscript. Nikhil Kumar: Concept, Data collection, Literature search, Editing manuscript, Approving final version of the manuscript. Himel Mondal: Concept, Data analysis, Visualization, Writing manuscript, Approving final version of the manuscript.

Example of conversation with ChatGPT 1

We asked ChatGPT to explain why fine needle aspiration cytology examination of the thyroid may not be helpful in diagnosing many of the thyroid lesions. The part of the answer is shown on this screenshot

Example of conversation with ChatGPT 2

We asked ChatGPT to explain why transfusion-related diseases are avoidable. The part of the answer is shown on this screenshot.
